# Not Every Country Can Absorb a Shock: Unequal Capacity to Withstand World Health Organization Aid Cuts

**DOI:** 10.1002/puh2.70208

**Published:** 2026-03-11

**Authors:** Animesh Ghimire

**Affiliations:** ^1^ Sustainable Prosperity Initiative Nepal Kathmandu Nepal; ^2^ School of Public Health Chitwan Medical College Chitwan Nepal; ^3^ School of Nursing Chitwan Medical College Chitwan Nepal

**Keywords:** aid cuts, donor exit, global health financing, health system shock, resilience, transition discipline

## Abstract

External health aid is contracting sharply, and this moment is often framed as a stress test of health system resilience. This perspective argues that it is equally a stress test of global health ethics and governance: When external actors withdraw from essential services, they are not merely responding to fiscal constraints—they are shaping who loses care. In 2025, the World Health Organization (WHO) projected a 30%–40% decline in external health aid to low‐ and middle‐income countries (LMICs) and urged governments to protect essential services, integrate programs into primary healthcare, and improve efficiency. Yet recent developments show that even well‐intentioned national reforms cannot fully absorb abrupt donor exits when core functions have long depended on external finance and coordination. Drawing on up‐to‐date evidence from Nepal and Afghanistan, this article shows how abrupt reductions in WHO and United States (US) support have simultaneously disrupted family planning, nutrition, immunization, community‐based care, and disease surveillance. In Nepal, the halt of US assistance exposed profound single‐donor dependence for contraceptive supply, nutrition programing, and elements of immunization financing—turning commodity gaps into system‐wide operational strain. In Afghanistan, a funding shortfall placed a large share of WHO‐supported facilities at risk of closure as measles and other outbreaks intensified, amplifying existing access barriers—especially for women and girls. These cases reveal a missing governance standard: There is no shared rulebook for how external actors should exit from essential services in fragile settings. This perspective proposes “transition discipline” as a practical global norm that links any reduction in support to (i) a time‐bound grace period, (ii) a publicly specified minimum protected service package, and (iii) a transparent, joint transition plan with clear responsibility. Transition discipline cannot eliminate all risk, but it can make inevitable cuts more predictable, accountable, and less detrimental.

## Graphical abstract

Abrupt cuts in external health aid are shown to destabilize multiple essential services simultaneously in fragile settings. Evidence from Nepal and Afghanistan reveals cascading disruption across family planning, nutrition, immunization, community‐based care, and disease surveillance. Transition discipline is proposed to require a grace period, a protected minimum service package, and a transparent transition plan during donor exit.

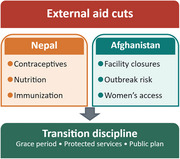

## 1 Introduction: One Global Message, Unequal Starting Points

In November 2025, World Health Organization (WHO) issued a warning regarding cuts to health financing, characterizing the situation as a shared challenge. It was anticipated that external health aid would decline significantly, prompting all governments to prepare in similar ways: safeguarding essential services, integrating vertical programs into primary healthcare, and making difficult funding decisions [1]. The rationale behind this message is clear: As external financial resources diminish, domestic systems must find ways to accomplish more with fewer resources. In health systems terms, abrupt aid withdrawal constitutes a “shock”—a sudden disturbance to financing, inputs, and coordination that can propagate across service delivery, governance, and population health over short time horizons [2]. However, this seemingly straightforward guidance conceals a deeper political and ethical dilemma. It presumes that all low‐ and middle‐income countries (LMICs) are positioned similarly, which is not the case. This unevenness is well established in the resilience literature, which conceptualizes health system resilience as the capacity to absorb, adapt, and transform in response to shocks and emphasizes that these capacities are distributed unequally across settings [3, 4]. When absorptive capacity is low, shocks can generate compound “operational burden” across multiple domains—financial strain, material shortages, workforce stress, quality erosion, and infrastructure limitations—rather than a single, isolated service failure [5].

At the same time, a parallel body of work on donor transition and sustainability planning—often focused on countries graduating from major global health initiatives—documents how funding cliffs can be mitigated through readiness assessments, co‐financing trajectories, and negotiated transition plans [6, 7]. Broader scholarship on global health financing and governance similarly highlights how fragmentation, earmarking, and politically contingent resource flows can undermine accountability and exacerbate vulnerability when priorities shift [8, 9]. Building on these debates, this article introduces “transition discipline” as a distinct contribution: A proposed governance norm that links exit decisions from essential services to explicit obligations (a time‐bound grace period, a minimum protected service package, and a publicly shared transition plan), rather than treating withdrawal as a purely technical or budgetary adjustment.

Ghana and Nigeria illustrate the first group. Ghana's government has removed a cap on transfers from the National Health Insurance Levy, injecting an estimated 3.4 billion Ghanaian cedis ($312 million) into the National Health Insurance Scheme in 2025 and creating additional space for claims, medicines, vaccines, and primary care [10, 11]. Nigeria has increased its federal health allocation and created a dedicated buffer to protect priority programs as external funds decline [12]. These examples illustrate that where fiscal space and administrative discretion are relatively greater, systems may be able to absorb or adapt to financing shocks without immediate, multi‐program service collapse—although such adjustments remain politically contested and uneven.

At the other end of the spectrum are countries such as Nepal and Afghanistan, where external support has long financed not only disease‐specific initiatives but also the basic functioning of primary care [13, 14]. Nepal and Afghanistan are selected as illustrative high‐dependence cases because both experienced contemporaneous, rapid disruptions to external support in 2025, and both operate under constrained fiscal and governance conditions that make abrupt exits more likely to exceed absorptive capacity and intensify operational burden. Comparable patterns have been observed beyond these two settings—for example, in donor transitions affecting human immunodeficiency virus/tuberculosis (HIV/TB) programs in Eastern Europe and Central Asia [15] and in sub‐national maternal and newborn health services in Uganda following donor withdrawal [16]—suggesting that the underlying governance problem is broader even when local manifestations differ. In these contexts, the manner in which WHO and donors choose to withdraw is just as critical as their guidance on the actions governments should take next. The subsequent sections intentionally emphasize urgent services currently under pressure in these two countries, illustrating how a global reduction in support translates into specific local crises. This article draws on publicly available sources, including WHO statements and situation updates, government documents, partner and procurement reports, peer‐reviewed literature, and corroborative reporting from reputable media outlets. Key figures and timelines have been compiled from these materials and cross‐verified across sources whenever possible.

## 2 Nepal: When Many Services Depend on One Donor

Recent reporting from Nepal reveals that the United States’ (US) halt of aid has not just caused a contraceptive shortage. It has shaken several pillars of the health system at once. In resilience terms, this abrupt funding interruption functions as a system shock to both financing and service‐support “inputs,” rapidly creating operational burden across commodity availability, budgets, outreach capacity, and the continuity of routine services [2, 5]. For many years, US aid to Nepal's health sector has flowed through both the treasury and non‐state channels. Estimates from the Ministry of Health and Population indicate that around 5 billion Nepali rupees ($34.31 million) a year were provided directly to government programs, with even larger sums channeled via nongovernmental organizations [17]. American support has been particularly significant in nutrition, immunization awareness, community health promotion, free treatment for TB and HIV, and a range of research and outreach activities [17, 18].

In 2024, the US allocated $72 million, approximately 10 billion rupees, for integrated nutrition and health programs in 498 municipalities across 48 districts over a 5‐year period. These initiatives are designed to combat stunting and wasting among children while also providing support for pregnant and lactating women [19]. National survey data indicate that the stunting rate has declined from 57.5% in 1996 to 25.5% in 2022; however, 8% of children still experience wasting [20]. Public health experts in Nepal caution that any setbacks in these programs could adversely impact the physical and cognitive development of the next generation [17].

Immunization has also relied on a mix of domestic and external finance. According to WHO figures for 2023, Nepal spent 3.95 billion rupees ($27.1 million) on regular immunization. Of that total, 1.95 billion rupees ($13.3 million) was spent on vaccines themselves, and the government covered roughly half the overall cost, about 540 million rupees ($3.7 million) of the vaccine bill, and a larger share of delivery expenses [17]. The rest depended on partners. The efficacy of these programs is integral to the nation's objective of reducing the under‐five mortality rate from 27 deaths per 1000 live births in 2022 to 20 by 2030 [21].

Since early 2025, the cessation of aid from the United States Agency for International Development (USAID) has resulted in three immediate and interconnected impacts on Nepal's health services. Together, these effects illustrate the concept of “operational burden” as a complex consequence of disruptions—encompassing simultaneous financial strain, material shortages, a reduction in service‐support capacity (including outreach efforts), and an increased risk to the quality and continuity of services [5]. The most visible has been the disruption of family planning. Facilities in many provinces have gone months without Depo Provera, intrauterine devices, and contraceptive implants [18]. At the same time, appreciation of the US dollar has increased the local currency cost of imported contraceptives, whereas the national family planning budget remains far below the estimated annual need of 540 million rupees ($3.7 million). Even after a rise to 100 million rupees ($686,226) in 2025, this leaves a gap of about 440 million rupees ($3 million) [18]. Additionally, USAID‐funded programs in private organizations have either shut down or reduced their scale, leading more clients to seek services at public facilities that are also out of stock.

Second, nutrition programs for children and pregnant women face uncertainty. The $72 million designated to support integrated nutrition efforts over the next 5 years is no longer guaranteed. Consequently, community‐based feeding, counseling, and monitoring activities in nearly 500 municipalities are at risk of being scaled back or discontinued [19]. Coupled with rising food prices and climate‐related disruptions, this withdrawal could impede or reverse progress in reducing stunting and exacerbate wasting among the most vulnerable populations [17].

Third, the broader package of community‐based services around immunization, maternal and child health, and family planning has lost a major source of operational funding. Nongovernmental organizations that relied on US grants for outreach and awareness campaigns have reduced activities or closed projects. Although the Government of Nepal has declared that it will not stop immunization or nutrition programs, senior officials acknowledge that domestic investment capacity is limited and that resources must be reallocated from other sectors if these activities are to continue at the current scale [17].

Nepal's situation cannot simply be characterized as a “contraceptive crisis.” It represents a complex interplay of multiple challenges affecting contraceptive supply, child nutrition, vaccine financing, and community‐based service delivery. Each of these sectors is affected by the same upstream decision to suspend US aid, a support system they all relied on. Comparable multiservice disruptions—often involving commodity stock‐outs, financing gaps, and loss of workforce or outreach capacity—have also been documented in other donor transition or exit contexts, underscoring that Nepal's experience reflects a broader governance vulnerability when exits are abrupt or weakly sequenced [6, 22]. However, there was no explicit mechanism in place to ensure continuity while the government sought internal resources or alternative partnerships. This fundamental oversight is precisely what the concept of *transition discipline* aims to address.

## 3 Afghanistan: Many Urgent Needs in a Collapsing Primary Care System

Afghanistan shows a similar pattern of multiple services under threat, in a context of even more severe fragility. On March 17, 2025, WHO cautioned that 80% of the essential healthcare services it supports in Afghanistan may close by June due to a funding shortfall [14]. As of March 4, 2025, 167 health facilities had already shut their doors, leaving approximately 1.6 million people without access to care [14]. The WHO reported that more than 220 additional facilities could potentially close in the coming months, putting another 1.8 million people at risk of losing services [14]. Other assessments indicate that if the worst‐case scenario unfolds, as many as 3.4 million Afghans could be deprived of necessary healthcare access [23]. Seen through a health system resilience lens, this is a compound shock: Abrupt financing contraction coincides with outbreak pressures and severe access constraints, rapidly overwhelming absorptive and adaptive capacities and generating operational burden across service continuity, surveillance functions, and workforce retention [2, 4, 5].

These closures are not limited to one type of care. They affect a wide range of urgent services. For instance, routine primary care, including management of common infections and chronic conditions, is disappearing in many districts. Clinics that once provided basic diagnosis, treatment, and referral are locked, without any clear notice to the communities [24]. Nutrition services for children and pregnant women, already strained by drought and economic collapse, are being scaled back [25]. Mental health and psychosocial support programs are shrinking. Disease surveillance is weakening as WHO‐led coordination structures lose staff and logistical support [14]. The cumulative impact of these disruptions illustrates the multifaceted challenges that resilience research identifies in the context of shocks. These challenges encompass concurrent pressures on financial resources, medical supplies and logistics, human capital, and information systems [3, 5].

At the same time, Afghanistan is facing concurrent outbreaks. In the first 2 months of 2025, more than 16,000 suspected measles cases and 111 deaths were reported [14]. WHO and other agencies have documented critically low measles immunization coverage: About 51% of children have received the first dose, and 37% have received the second. Malaria, dengue, poliomyelitis, and Crimean–Congo hemorrhagic fever are also circulating [14, 26]. Without functioning primary care facilities, it becomes much harder to detect and respond to these diseases.

The social environment makes this collapse even more dangerous. Restrictions on the movement, education, and employment of women and girls have sharply reduced the number of female health workers and made it harder for women to travel for care [27]. Many of the facilities now at risk of closure were among the few spaces where women could see a female health provider [28]. When these sites close without replacement, barriers to access multiply.

As in Nepal, these outcomes were predictable. WHO, donors, and humanitarian agencies knew how many services depended on external money and coordination. They knew that measles coverage was low and that outbreaks were likely. They knew that women's access to care was already constrained. Yet the funding reduction has occurred without a “binding” requirement to preserve a defined minimum package of services during a transition period.

## 4 The Problem With Resilience Talk

In both Nepal and Afghanistan, the WHO's guidance to “protect essential services” and “integrate programs into primary healthcare” is technically sound but lacks political depth [1, 14]. In the health systems literature, abrupt aid withdrawal is a form of “shock”—a sudden disturbance to financing and operational inputs that can cascade across routine functions and outcomes [2, 29]. Resilience frameworks conceptualize responses to shocks through absorptive, adaptive, and transformative capacities, mediated by governance and legitimacy as much as by technical capability [3, 4]. A core insight from this work is that shocks often generate “operational burden” across multiple domains—financial strain, commodity and logistics disruptions, workforce stress, and weakened information/surveillance—rather than a single, isolated program failure [5]. It emphasizes actions that ministries of health should take during periods of significant funding, staffing, and operational capacity losses. Read through resilience terminology, these recommendations largely target absorptive and adaptive responses (e.g., protecting routine functions and integrating fragmented delivery platforms), but they say less about the governance of the shock itself when it is produced by external exit decisions.

Moreover, it lacks clarity regarding the necessary modifications in strategy that WHO and funding entities must adopt when they opt to withdraw their support. It is crucial to delineate the minimum continuity obligations that these stakeholders are willing to accept in such scenarios. This discrepancy is significant. When external actors have played a pivotal role in financing and shaping essential programs for years, their decision to withdraw is not a neutral event; rather, it profoundly influences local realities. The concern here is therefore not with resilience as an analytic concept, but with a strand of “resilience talk” in global policy that can treat externally generated shocks as inevitable and locate responsibility primarily in domestic adjustment. Critical scholarship has warned that resilience narratives may depoliticize vulnerability, narrow democratic priority‐setting, and obscure power and accountability in global health governance [30, 31]. Discursive analyses of “resilience” and “self‐reliance” in global health also show how competing ideologies shape where responsibility is assigned between global actors and national systems [32], reinforcing calls for conceptual clarity so that “resilience” does not become a catch‐all slogan that weakens accountability [33, 34]. Framing the situation merely as a test of domestic resilience risks transforming a global policy choice into a narrative of local blame. A more honest and constructive perspective is to analyze the cessation of essential services as governance events in their own right.

The relevance of donor transition literature is particularly pronounced in this context. Current transition frameworks focus on sustainability planning, co‐financing pathways, and negotiated handovers. However, they tend to assume orderly timelines and offer limited enforceable standards for the sequencing of exits. This is especially true when reductions are sudden, politically motivated, or take place in fragile environments, where ensuring a minimum package of essential services becomes critical [6, 7]. Transition discipline is articulated as a complementary framework to resilience thinking, delineating both ethical and practical responsibilities for external stakeholders in the context of exit‐related disruptions. By emphasizing foreseeability, shared accountability, and transparency, this approach aims to clarify these principles rather than leave them to implicit understanding. This encapsulates the core idea behind “transition discipline.”

## 5 Transition Discipline: Linking Exit Decisions to Ethical and Practical Obligations

Transition discipline is a proposed norm that would bind external funders, including WHO when it plays an operational role, whenever they plan to reduce or end support for essential health services. It is intended to enhance—not substitute—existing health system resilience frameworks. Although resilience concepts illustrate how systems can absorb, adapt to, and transform in response to shocks, transition discipline clarifies the governance responsibilities of external actors whose funding decisions can create or exacerbate shocks and their associated operational challenges [2, 4, 5]. It has three elements. The first element is a grace period. This is a clear, time‐limited commitment, usually between 12 and 18 months, during which a minimum level of essential services will be maintained while domestic systems adjust. The length of the grace period is not arbitrary. It reflects the time needed to revise budgets, negotiate new contracts, arrange co‐financing, and reorganize delivery without abrupt service loss. In effect, it functions as bridge financing and a planning horizon that stabilizes procurement and operations—features repeatedly identified as prerequisites for responsible exits and sustained impact [22, 35].

The second element is a minimum package of protected services. This package must be specified publicly at the start of the grace period. In settings like Nepal and Afghanistan, it would include routine immunization, antenatal and postnatal care, basic delivery care where feasible, contraception, nutrition support for children and pregnant women, treatment of common childhood illnesses, selected essential medicines, and basic disease surveillance. Making the package explicit converts broad commitments to “protect essential services” into a verifiable benchmark, enabling public scrutiny of whether withdrawal is compatible with minimum population health protection.

The third element is a joint transition plan. This plan sets out who will finance and manage the minimum package during the grace period and how responsibilities will gradually shift to the national system or to alternative partners. It must cover financing, procurement, logistics, workforce retention, data, and accountability. It should be made public so that citizens, parliaments, and civil society can see what is being promised and by whom. Evidence from donor transitions and co‐financing experiences suggests that such plans are most credible when they include explicit milestones, shared financing assumptions, and an agreed monitoring mechanism that involves both health and finance authorities [36, 37].

Existing donor transition frameworks, commonly referred to as “graduation,” sustainability planning, or transition readiness, prioritize the mobilization of domestic resources, enhancement of institutional capacities, and the establishment of negotiated handover processes. These approaches underscore the importance of creating a sustainable financial ecosystem and robust institutional frameworks to facilitate a seamless transition [6, 7, 38]. At the same time, global health financing scholarship highlights persistent problems of fragmentation, earmarking, and weak accountability that complicate orderly transitions and can incentivize abrupt, donor‐driven exits [8, 9]. Transition discipline builds on these insights but adds a distinct governance claim: Regardless of why funding is reduced (income‐based graduation, political reprioritization, or fiscal contraction), external actors should be bound to (i) a defined grace period, (ii) a publicly specified minimum service package, and (iii) a public, jointly owned plan that sequences withdrawal and assigns responsibility.

Transition discipline is feasible only if it is made operational through concrete instruments. Four mechanisms are plausible. First, it can be embedded as a default clause in grants, compacts, and procurement agreements (including co‐financing arrangements), specifying minimum‐package protection and a transition plan as conditions of exit rather than optional “best practice” [37]. Second, it can be financed through pooled “transition” or bridge windows—funded by a small top‐slice of major grants or by multi‐donor risk‐sharing—to cover the grace period when individual donor lines are cut [8]. Third, independent monitoring can be institutionalized through public reporting of transition plans, milestones, and service‐coverage indicators, drawing on evaluation approaches recommended in the transition literature [6, 36]. In fragile or politically constrained environments where governments are unable or unwilling to collaborate, the “joint” plan can outline alternative delivery mechanisms, such as third‐party implementers. Fourth, it is essential that this plan maintains a commitment to transparency regarding financing, coverage targets, and safeguards for vulnerable populations—concerns that have frequently emerged in analyses of abrupt transitions and high‐dependence contexts [6, 38].

Transition discipline does not demand that donors maintain past levels of spending. It does not prevent WHO from reallocating resources when its own budget is cut. It does insist that exits from essential services are sequenced, deliberate, and transparent, and that predictable harm is minimized. Its core purpose is to ensure that unavoidable fiscal constraints do not translate into avoidable service collapse.

## 6 What Transition Discipline Could Have Changed in Nepal and Afghanistan

In Nepal, transition discipline would have required WHO, the US government, and other major partners to announce clearly that funding for nutrition, immunization, and family planning programs would be reduced or suspended through a publicly released transition notice specifying the timing of reductions, the grace‐period duration, and the minimum protected service package. Together with the Government of Nepal, they would have estimated the monthly cost of maintaining a basic set of services in these areas and committed to financing that cost for at least 12 months while domestic resources were mobilized and procurement was completed. To make this commitment enforceable rather than aspirational, it could be codified through amended grant agreements, memoranda of understanding, or time‐bound bridge‐financing arrangements that specify deliverables (e.g., commodity quantities, outreach coverage, and reporting requirements), consistent with lessons from donor transition planning on the importance of explicit timelines and shared financing assumptions [6, 7]. By stabilizing procurement and retaining outreach capacity during the grace period, transition discipline would reduce the “operational burden” of the shock—limiting preventable commodity interruptions, workforce attrition, and knock‐on effects on service quality and surveillance [5].

The Government of Nepal, in turn, would have been expected to reallocate funds from lower priority activities, as its own minister of health has suggested, and to seek additional support from other partners [17]. Those domestic reallocations and partner commitments would be documented in the public transition plan, enabling oversight by parliament and civil society and reducing the likelihood of “silent” rationing [39]. The grace period would not have prevented all disruption, but it would have made months‐long contraceptive stock‐outs and the abrupt stalling of nutrition programs in hundreds of municipalities much less likely. If domestic replacement financing still fell short, transition discipline would at minimum require transparent disclosure of the gap and a jointly agreed contingency approach (e.g., prioritizing core commodities and outreach for the highest need areas), rather than ad hoc service collapse [6].

Applied to Afghanistan, transition discipline would have required WHO and other donors to identify a network of priority facilities that could not be allowed to close during the grace period because of the populations they serve and the lack of alternatives. Selection of this network would be guided by explicit, publicly stated criteria (e.g., catchment population, remoteness, outbreak risk, maternal services, and the availability of female providers), drawing on evidence that donor transition “prioritization” processes can generate avoidable harm when criteria and decision‐making are opaque [39]. Funding decisions would have been structured around keeping those facilities open, maintaining vaccination and basic care, and supporting mobile teams for areas where fixed sites were no longer viable. It would also have required explicit efforts to keep female health workers in post and visible, including negotiations with local authorities where necessary. Where national authorities are unable or unwilling to assume responsibility, the grace‐period minimum package could still be maintained through time‐limited contracts with implementing partners (with independent monitoring and public reporting), reflecting documented transition risks when core functions—logistics, workforce, outreach, and data—are not secured during handover [6, 40].

The difference is not purely theoretical. Had such a norm been in place, closure of services would still have occurred under pressure, but not at the speed and scale now observed, and not with so little protection for the people most at risk. Similar vulnerabilities—financing gaps, loss of outreach and staffing capacity, and service discontinuities driven by nontransparent or weakly sequenced transitions—have been documented in other donor exit contexts, including President's Emergency Plan for AIDS Relief (PEPFAR) transition‐related geographic prioritization in Kenya and Uganda and sub‐national maternal and newborn health programing after donor transition in Uganda [16, 39].

## 7 Limitations and Implications for Global Health Policy

This article draws on well‐documented recent developments in Nepal and Afghanistan to illustrate how current funding cuts are playing out in countries with limited capacity to absorb them. It does not cover all LMICs, nor does it attempt a systematic cross‐country comparison. Countries such as Ghana and Nigeria are mentioned only to show that domestic adjustment is possible when certain conditions are met, not as simple models to copy.

The concept of “transition discipline” is normative rather than empirical. It raises implementation questions, including who would enforce such a standard and how grace periods would be financed. Feasibility depends on operational mechanisms: Enforceability could be strengthened by embedding minimum‐package and grace‐period requirements within grant agreements, transition compacts, and procurement arrangements, paired with a publicly available transition plan and independent monitoring/reporting to clarify “who is accountable to whom, and for what” in funding exits. Financing the grace period amid donor contractions would require risk‐sharing instruments—such as pooled bridge windows or contingency mechanisms—that reduce incentives for abrupt unilateral exit and address persistent governance challenges in global health financing, including fragmentation, earmarking, and weak downward accountability. In contexts where host governments are unwilling or unable to cooperate, transition discipline would still require transparent specification of who will finance and deliver the minimum package during the grace period (including time‐bound third‐party delivery arrangements where necessary), consistent with calls for clearer “investment standards” and safeguards against avoidable system harm from poorly governed external financing. However, the cases of Nepal and Afghanistan show that the absence of such a norm has real, measurable consequences for contraceptive access, child nutrition, vaccination, primary care, and outbreak control.

For WHO and its partners, the implication is clear. While providing technical guidance to governments on how to address funding cuts is essential, it is not enough on its own. Global health stakeholders must also pledge to manage their withdrawal from essential services with careful discipline. Operationalizing *transition discipline* would mean treating donor exit as a governed process—subject to explicit standards, public documentation, and accountability mechanisms—rather than as an administrative endpoint [41, 42]. Adopting a framework of transition discipline represents a tangible approach to achieving this goal.

## 8 Conclusion

The ongoing reduction in external health aid serves as a significant stress test for the resilience of health systems. The situations in Nepal and Afghanistan illustrate that this is also a critical examination of global health ethics. Rather than restating the well‐established observation that countries vary in resilience capacity, this article's central contribution is to document how abrupt aid withdrawal can produce multiservice harms across family planning, nutrition, immunization, community‐based care, and disease surveillance and to propose transition discipline as a minimum governance and ethical standard for managing exits from essential services. When WHO and major donors withdraw support from essential services in fragile settings, their actions extend beyond mere technical responses to fiscal pressures; they represent political choices that dictate who loses access to contraception, who misses vaccinations, which children endure hunger, and which pregnant women are compelled to undertake perilous journeys in search of care. Adopting transition discipline as a global norm will not reverse the cuts already underway. It would, however, change how future cuts are made by requiring that they follow clear rules of foreseeability, shared responsibility, and transparency. This proposal aligns with wider debates in global health financing about sustainability, donor responsibility, and equity in an era of contraction, and it offers a practical governance response to ensure that fiscal retrenchment does not translate into avoidable service collapse. In an era of shrinking budgets, this is no longer optional. It is the minimum ethical standard for exiting from essential services, and it is the standard against which WHO and its partners should now be judged. Ultimately, abrupt donor exits constitute a health system shock that tests absorptive and adaptive capacities and generates operational burden; transition discipline is intended to reduce predictable harms by governing the timing, minimum package, and accountability of exit decisions.

## Author Contributions

Conceptualization: Animesh Ghimire. Formal analysis: Animesh Ghimire. Investigation: Animesh Ghimire. Writing – original draft preparation and writing – review and editing: Animesh Ghimire. The author approved the final version to be published.

## Funding

The author has nothing to report.

## Ethics Statement

The author has nothing to report.

## Conflicts of Interest

The author declares no conflicts of interest.

## Data Availability

Data sharing is not applicable to this article as no new data were generated in this study.
